# An ethnographic observation study of the facilitator role in an implementation process

**DOI:** 10.1186/s13104-017-2962-5

**Published:** 2017-11-28

**Authors:** Irén Tiberg, Kristofer Hansson, Robert Holmberg, Inger Hallström

**Affiliations:** 1 Department of Health Sciences, Lund University, 221 00 Lund, Sweden; 2 Department of Arts and Cultural Sciences, Lund University, Lund, Sweden; 3 Department of Psychology, Lund University, Lund, Sweden

**Keywords:** Implementation process, Facilitator, Change agent, Role

## Abstract

**Background:**

Even though the importance of a facilitator during an implementation process is well described, the facilitator’s role is rarely problematized in relation to the organizational context in terms of power and legitimacy; themes which have recently been brought to the fore when studying change in health care organizations. Therefore, in this article, we present a qualitative study with the aim of identifying key aspects of the experience of being in a facilitator role. The data collection involved ethnographic fieldwork encompassing observations and field notes, as well as two qualitative interviews with the facilitator. The data were analysed using a phenomenological hermeneutical method in order to formulate thematic aspects of the implementation process. The study was conducted in southern Sweden between January 2013 and August 2014.

**Results:**

One main theme, “walking a tightrope”, and four sub-themes, all of which involved balancing acts of different levels and different ways, were identified. These included: being in control, but needing to adjust; pushing for change, but forced to stand back; being accepted, but dependent; and being reasonable, but culturally sensitive.

**Conclusion:**

Instead of listing the desirable qualities and conditions of a facilitator, this study shows that being a facilitator can be described more completely by applying the concept of role, thus allowing a more holistic process of reflection and analysis. This in turn makes it possible to move from the reactive stance of balancing to a more proactive stance of negotiating.

## Background

The implementation of evidence-based interventions constitutes a basis for the development of health care and social services [1–4]. However, clinical implementations are often complex, involve many mechanisms and are social processes that are communicated over time through specific channels amongst members of a group, in order to reach a state of mutual understanding [5]. Implementation can be studied as a procedure and/or a process of change, i.e. something in which the ambiguities of the implementation process become visible and can be focused upon, which is central when the implementation is to be transferred between different settings [6–8]. Even though the importance of a facilitator to an implementation process is well described, few studies focus on the challenges the facilitator faces during the process and the impact this has on the process.

Harvey et al. [9] presented a conceptual analysis of the facilitator role, seeing it broadly as an example of a change agent role, with other examples of change agent roles being discussion opinion leader and educational outreach visitor. They begin with the development of a multidimensional framework in which successful uptake of research evidence is determined by the quality of the evidence, the qualities of the context (leadership, monitoring, feedback and culture) and the facilitation provided. Harvey et al. [9] suggest that facilitators are, “individuals with the appropriate roles, skills and knowledge to help individuals, teams and organizations apply evidence into practice” (p. 579). When the purpose of facilitation is to achieve goals, the role is largely concerned with providing practical help and support. When facilitation has a more broad focus on the development and empowerment of individuals and teams, there is at least an equal emphasis on the development of enabling processes or relationships [9]. The authors suggest that purpose, roles and skills in relation to facilitation can vary considerably and could be viewed as continua on which different approaches to facilitation can be mapped.

A recent review [10] of studies described change agents (to use the term broadly and include facilitators) and evidence-informed health care. One important factor found is that change agents are embedded in the context and accessible. Important characteristics of the change agent are being organized and culturally compatible in terms of their established connections with the target group. The change agent needs to be perceived by others as having expertise and as being credible in order to establish respect within the target group. In addition to a variety of characteristics of the change agent, the organization needs to be supportive and the change agent role seen as important and adequately resourced. Another discussion of the change agent role has increased the attention devoted to political issues in organizations and the need to take power into considering [11]. Other contributions highlight the complexities of organizational change and identify not only challenges for change agents, but also new strategies and modes of operation that have arisen in response to the emergence of new structural conditions and new strategies of change. Examples of these are active efforts not only to span existing boundaries, but also to redefine them in order to support development—boundary shaking [12]; radically distributed change agency in health care organizations [11], how practices of boundary organization help to accommodate political and professional tensions when developing and introducing new technologies in a health care context [13]. Balogun et al. [12] argue that the mainstream or functional conceptualization of change agents and change agency has severe limitations as it does not consider how the context a medium for the agency of change agents, but also, to a large extent, structures the various forms of change agency. They also draw attention to the limited treatment of power in the change agent literature.

The study of organizational roles has been approached from sociological perspectives [14] and from more psychological viewpoints. Reed and Bazalgette [15] argued that the role concept is often used in a way that emphasizes how others define a role for somebody—it is prescriptive and relatively static (hence the ever-growing lists of different roles for change agents and managers)—and that role and person tend to become separated. According to Reed and Bazalgette [16], a role is the outcome of an on-going dynamic effort at the intersection between person and system. The role is made possible when a person brings his or her capacities and desires into activities that are aligned with the purpose of a system by utilizing the resources available in that system. “To take a role implies being able to formulate or discover, however intuitively, a regulating principle inside oneself which enables one, as a person, to manage one’s behaviour in relation to what needs to done to further the purpose of the system within which the role is to be taken” (p. 46). This intersection between person and organization is developed further by Krantz and Maltz [17] who describe how a role is something that is partly actively taken by a person and, to some extent, given to them by the organization and the social context. The role also has aspects that are task-oriented and aspects that are more sentient and emotional and thus less explicit. The process of discovering one’s role or learning to act effectively in a role evolves through active experimentation in relation to the given aspects of the role and a gradual increase in one’s understanding of the system—in terms of both its explicit and its implicit or more sentient aspects.

A number of reviews of change agency and facilitation in nursing/health care identify tasks, skills and conditions for successful facilitation [9, 10, 18]. Accordingly, the facilitator role is rarely problematized in relation to the organizational context in terms of power and legitimacy; themes which have recently been brought to the fore in studies of change in health care organizations [9–13, 18, 19]. Furthermore, the literature concerning facilitation, change agency and the facilitator role mostly describes sets of skills and personal qualities. There are very few examples of the experience of being a facilitator or of taking on the facilitator role and what the process of facilitation might be like. Hence, in this article, we present a qualitative study based on a single case of one facilitator in a specific context with the aim of identifying key aspects of the experience of being in a facilitator role.

## Methods

The study was approved by The Regional Ethical Review Board in Lund, Sweden (LU 2013/326). Verbal information about the study was provided regularly to all members of the diabetes teams and they were asked for consent related to the ethnographic observations.

Data collection was performed when implementing hospital-based home care (HBHC) for children newly diagnosed with diabetes at a university hospital in southern Sweden. HBHC was defined as the delivery of hospital care to patients at home [20]. The study was part of an implementation study based on the Medical Research Council’s (MRC) framework for complex interventions [21, 22]. We have previously described the development of HBHC [23] and evaluated the effectiveness and cost-effectiveness of HBHC in comparison with the traditional hospital-based care [24–27]. Our results showed that HBHC was a safe and feasible way of caring for the child and the family. In this article, the implementation of HBHC into routine care is described.

The university hospital had recently been created through the amalgamation of two hospitals that are situated in the two largest cities in the region. The paediatric department was therefore divided between two local units, each unit having a diabetes team consisting of paediatric nurses specialised in diabetes, paediatricians specialised in paediatric diabetes, dieticians and social workers. The active parts (i.e. key components expected to have an effect) of HBHC were defined as (1) an individualised learning process through daily, supportive interaction between the family and a diabetes nurse, (2) a “home-like” environment where families could practice the management of diabetes with concurrent support and (3) increased support in the form of three home and/or school visits by the diabetes nurse upon discharge, in addition to the regular diabetes check-up visits and increased access to the diabetes nurse by telephone.

### The facilitator and the facilitating strategy

The facilitator, or the change agent as Rogers name the role [5], is ideally a professional who has influence in the system in which an innovation is to be implemented, and who is based externally to the system. Therefore, the facilitating support was carried out by a person who is well known to the diabetes teams and familiar with the content of HBHC (first author: IT). The facilitator worked as a paediatric diabetes nurse in both units until March 2013, but was primarily assigned to one of the units. From April 2013, the facilitator was employed outside of the health care system and was part of the research project. Previously, the facilitator was responsible for the RCT that evaluated the effectiveness of HBHC and hospital-based care in one of the units between 2008 and 2013. As it was the first time the facilitator was acting in the role of an external facilitator, she received mentoring and regularly supervision from an experienced psychologist.

Everett Rogers’ framework of diffusion of innovations [5] was the theoretical starting point for the facilitator and the planned facilitating support; consequently this also guided how the support was implemented. The framework views diffusion as a social process in which participants create and share information with one another in order to reach a mutual understanding. The way in which the characteristics of the innovation are experienced by the individuals in the system therefore has an impact on the diffusion of the innovation. The characteristics of importance to the diffusion of the innovation are, according to Rogers [28], its relative advantage, its compatibility, its complexity and how understandable it is to each team member. Other characteristics of importance are how familiar team members are with the innovation and how visible the results of the use of the innovation are to others. Within the theoretical framework, seven roles have been identified for the facilitator in the process of introducing an innovation in a system [5]. The different roles that guided the facilitating support included developing a need for change in the group—establishing an information exchange relationship by being perceived as credible, competent and trustworthy and by empathizing with the group’s needs and problems. Further roles involved identifying problems in order to determine why existing options did not meet team members’ needs—creating intent to change and translating this intent into action by motivating the team members’ interest in the innovation. Through interpersonal networks, the facilitator sought to influence individuals’ behaviour. Later in the process, the facilitator’s role was to stabilize the new behaviour or procedures by providing reinforcing messages to the team members who had adopted them. The end goal was to facilitate each team member’s transition from being dependent to being trusted and independent.

**Fig. 1:**
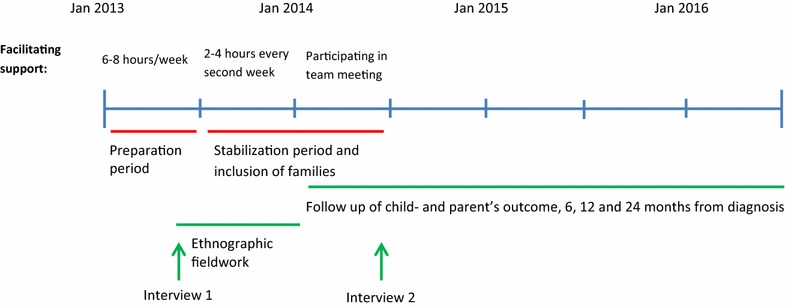
Flowchart with an overview of the study with data collection and summarizing the facilitating support in the different periods. The data collection reported in this article included ethnographic fieldwork and two qualitative interviews

The implementation process began in January 2013 and started with facilitating support being provided for one and a half years (Fig. 1). The facilitating support included two periods: a 6 month preparation period from January to June 2013 that aimed to prepare the diabetes teams for the new procedures, followed by a 1-year stabilization period that aimed to support and stabilize the new procedures. During the preparation period, the facilitator spent 6–8 h each week at each unit in order to discuss procedures and approaches with the team members and to provide them with support and individual feedback after they meet with families individually. For the first 5 months (August to December 2013) of the stabilization period, the support involved 2–4 h every other week at each unit, in addition to participation in team meetings. Over the course of the next 7 months (January to June 2014), the support was gradually reduced and involved participation in team meetings every other week.

### Data collection

An ethnographic method was used for the data collection and this involved ethnographic fieldwork and two qualitative interviews with the facilitator (Fig. 1). The ethnographic fieldwork, conducted by the second author (KH) in the period from April 2013 to December 2013, involved making observations and field notes during six diabetes team meetings. Each team meeting lasted for approximately 3 h. The field notes were transcribed by the ethnologist (KH) directly after each observation in order to create a coherent text. The transcribed field notes from the observations were read by the facilitator, who also made reflective notes on the transcriptions, describing her role as a facilitator and providing background knowledge for clarification purposes. Two qualitative interviews with the facilitator were carried out by KH. The facilitator was asked to describe her experiences of specific situations described in the transcriptions of the observations. The first interview took place during the preparation phase and the second interview was conducted at the end of the stabilization period. The interviews were recorded and the recordings transcribed by an independent person not involved in the study.

### Data analysis

Essential data was elucidated as it was lived in human experience by using a phenomenological hermeneutical method inspired by Ricour, as described by Lindseth and Norberg [29]. The focus in the analysis was on identifying key aspects of the experience of being in a facilitator role. The data analysis process included several different layers that originated from researchers with different backgrounds and from different academic disciplines, including medical ethnology, nursing, and psychology.

The phenomenological hermeneutic analysis included three steps: naïve reading, structural analysis and comprehensive understanding (interpreted whole) [29]. Three of the four authors individually read the transcribed observations and transcribed interviews several times in order to gain an understanding of the text as whole and grasp the overall meaning. During this step, notes were written in the margins and phrases and quotes that seemed to reflect the study’s aim were marked. In the second step, a structural analysis was performed that started by identifying meaning units that reflected an essential meaning of the lived experience. Keeping the naïve understanding in mind, the authors reflected upon clusters of meaning units and the meaning was interpreted by asking what they revealed about the facilitator role. Thereafter, the authors sought to identify and formulate preliminary themes, expressed in everyday words, for each interview and field note. The first author with the facilitator role was excluded from the first and second steps of the analysis. In the third and final step, all four authors summarized the preliminary themes and sub-themes and compared and reflected on them in relation to the research question and the study context. The authors agreed upon the formulation of a comprehensive understanding that is described in one main theme summarized in a metaphor and for sub-themes.

## Results

The main theme identified in the result, “walking a tightrope”, is formulated as a metaphor and thereby frames the key experiences that were interpreted. Thereafter, each sub-theme is presented individually: being in control, but needing to adjust; pushing for change, but forced to stand back; being accepted, but dependent; and being reasonable, but culturally sensitive. In the interviews, the facilitator referred back to describing her feelings of inadequacy. Despite having thorough theoretical knowledge of the implementation process and the role, the facilitator still felt insufficiently prepared. In one of the interviews, she described the facilitator role as like, “having a pasture full of wild horses that you have to control and each one is running in a different direction with its own motivation”. In this article, we take this kind of metaphor as our starting point in order to understand and describe the facilitator role and argue that thinking about the social world is not possible without metaphors [30, 31]. We use these metaphors to try to open up new and creative ways to discuss and understand what it means to be in a facilitator role.

The main theme, “walking a tightrope”, illustrates the demanding process in which the facilitator guides a group of staff towards a set goal. When the theory was compared with the practical experience of being a facilitator, there was an imbalance in terms of their complexity. The facilitator had to balance and walk a tightrope; she had walked this way before and was aware of the conditions caused by bad weather and fickle winds. These aspects challenged the balance, but the metaphor also created an understanding of the opportunities to adapt and gain better balance. Like a downhill skier, the facilitator developed balancing techniques to use when the changing winds were too strong and she needed to reduce air resistance and friction. At other times, she needed the friction to stop the process and stand up for her cause. In this position, the imbalance could quickly remind her of the position she held in the group. It was a balancing act similar to how a child experiences a seesaw; sometimes the person sitting on the opposite side is just too heavy and at other times you place yourself too close to the centre and cannot take advantage of the leverage effect. Accordingly, it involved finding a balance by moving back and forth on the seesaw, depending on the conditions on the opposite side.

### Being in control but needing to adjust

A central sub-theme was how the facilitator at the same time as she had control over the meetings, needed to adjust this control in relation to the group and continuously consider and moderate the behaviour of the team members towards each other. At the same, time the facilitator needed to relate this to the actual procedures of the clinical settings and the goals of the changes to procedures.

It was the job of the facilitator to present the anticipated changes and approaches to procedures, as well as to create opportunities for the change process and the stance held by each team member in relation to the implementation of new procedures. Written agenda for meetings were introduced and emailed to the group prior to meetings, giving the team members an opportunity to provide feedback and enhancing the possibility of fitting short structured meetings into a busy workload. The meetings were seen as an opportunity to discuss other issues that were of concern to the group but, perhaps not relevant to the implementation process. In these situations, the facilitator had to adapt her presentation to the interests of the group, allowing them the freedom to control or run the meeting and enabling the group to move forward according to the group’s needs and the situation. If the facilitator met with resistance from the group, an imbalanced situation was created and the sudden stop needed to be parried. The facilitator role did not solely involve highlighting the needs of the group, but also involved balancing the group’s need to push the change process forward when possible, which became two incompatible perspectives.

Within the context of this implementation process there were many actors with different wills, wishes and intentions. The example in Textbox 1 demonstrates how quickly the balance in a group can change and make the discussion shift in another direction than planned and how difficult it can be to guide the discussion. Examining the situation described reveals that it was important for the group members to discuss the current documents in relation to the implementation, the members would not give up using these documents and there was a further need for discussion. The balance between allowing the group to discuss freely or to be more controlling can be contemplated in terms of the metaphor of the seesaw, with the facilitator moving back and forth depending on the conditions of the situation.

#### Textbox 1

On one afternoon in May 2013, the group was gathered in a large room, sitting behind tables set out in a large U-formation. The facilitator was about to present the forthcoming procedure changes and wanted to move on from the first topic on the agenda which was not directly related to the implementation process, when one of the leaders of the group made further comments related to another document—a checklist relating to the current procedures. The current procedures symbolized what the facilitator wanted to move away from. The facilitator said “In conclusion we will continue to work with these [current] documents…” and even if the leader believed that the document needed more discussion, the facilitator tried to aid progression with a quick comment. This was unsuccessful; instead, several team members continued to comment on the checklist. The group discussions changed to a topic that was obviously of great concern to many in the group and everyone started talking to the person sitting next to them. This example illustrates how the facilitator could not rely on being the one in control of the meeting; she had to relate to the dynamics within the group and balance her intensions and the wishes of the group.

The balance between control and adjustment became an example that was used to understand how the facilitator related to the situation and to leaders of the group. There was a balancing act that involved reflecting on how to weigh up the importance of leadership in terms of its positive impact on the implementation process against the risk that the leaders would take over control of the situation and the risk of not keeping to the meeting’s agenda.

### Pushing for change, but forced to stand back

The subtheme focus how the facilitator was pushing for change in the group, but for different reasons was forced to stand back and accept the group’s decisions. The balance between control and adjustment was based on the facilitator’s goal that the group members move towards accepting the changes to procedures. If the group demonstrated reluctance, the facilitator was forced to stand back. The facilitator needed to choose a path that took into account possible resistance and allowed the procedures to be modified in accordance with the group’s wishes. In the previously described situation (Textbox 1), it could be stated that there was resistance from parts of the group to giving up the checklist. By raising the discussion of the checklist, some of the team members might have pointed out that they would not accept the changes to procedures as they were planned. Adaptation and resistance can also be applied to understanding the facilitator’s relationship to the group because she could also choose to either adapt suggested modifications to the procedures or to resist them if they were not compatible with important principles of the changed procedures.

In the situation described in Textbox 2, the digital scale became a suggestive object that enabled the team members to see things from the family’s perspective. The discussions of weighing food also made the team members’ attitudes towards the changed procedures clear to the facilitator, and these attitudes did not balance well with the facilitator’s wishes.

#### Textbox 2

In December 2013, a situation took place when the team members had been to a nationwide meeting at which clinical procedures were discussed. A team from another part of the country stated that they had started with carbohydrate counting, meaning that families with a child diagnosed with diabetes needed to weigh most of the food the child would eat. The facilitator, who also attended the meeting pointed out that the method was emphasized as very useful, but the facilitator was also critical of the method: “is this the picture we want to paint for families living with type 1 diabetes?” One of the leaders of the group did not seem to pay attention to the argument and said, after some discussion, “I definitely think that we should implement a ‘light’ version, that we recommend that families should weigh the food in the beginning”. The person continued by saying “I assume that it cannot be too expensive to buy scales for each unit?” This was a decision that went against the important principles of the new procedures; care based on the family’s needs. The defusing way of accepting and resisting the decision continued during the group discussions of scales. One of the team members was sceptical, saying “we have some families who will get stuck and weigh all food”. Weighing food might put treatment at risk by hindering everyday life to a greater extent than necessary. Some team members emphasized more positive aspects, “I have friends that always weigh and measure what the child drinks and eats, and they think it is fun”. The diverged discussion opened up a possibility for the facilitator to conclude with advantage by pointing out that “perhaps it is not our decision; we can leave it up to the families to decide for themselves”.

### Being accepted, but dependent

The facilitator needed to be accepted by the group in order to succeed with the implementation, but at the same time she became dependent of the group. In order to understand the facilitator’s position in terms of adaptation and resistance in the given examples (Textboxes 1, 2), it is also necessary to understand the position of dependence the facilitator had in relation to the group; for example, the facilitator’s dependency on the group was due to this being, until recently, her place of work, thus creating special circumstances that she needed to consider. In this situation the facilitator knew the team members, was familiar with previous conflicts, informal leaders, etc. The way the position of dependence or independence developed related to the situation. When interviewed after the facilitation period, she described the positions in relation to the group:

“I feel like I’ve been walking a tightrope for a year and a half, and have tried to… In the role of a researcher, I wanted to hold on to the evidence; what we have evidence for. In the role as a paediatric diabetes nurse, I understand the team members’ situation very well and in the role of a facilitator, I tried to push the process forward, but at the same time, it felt very troubling that I didn’t have the role of a natural leader. I have a long history of not being the leader in the group—the other people are those with the leadership roles. It’s been a situation in which I’ve been conflicted; trying to take on the role of leader, which was completely unnatural to me.”

This quotation shows different and conflicting roles to which the facilitator felt she had to relate during the implementation process. However, it was also a perspective on the past and thereby what had been experienced. The present perspective—at the time of the interview—was described as an act of balancing the different roles: researcher, leader but also former nurse. All these roles were related to different expectations, from both the facilitator herself and the team members. The facilitator’s own perceptions of how the team members related to her were central. The facilitator’s found the expectation that she would be a leader troublesome as it did not come naturally to her and she was not permitted to assume the lead role in the group. The conflicting situation was a mixture of having the leading role in the implementation process, while also being dependent on formal and informal leaders in the group. It became evident in the previous examples that it was individuals in a leading position who raised suggestions or arguments that were in conflict with the implementation of the new procedures that the facilitator was tasked with facilitating. The facilitator was not independent in her role as facilitator; she was instead strongly dependent on the leaders of the group and needed to strike a balance between, on the one hand, not criticizing these leaders and becoming a threat to their leading positions in the group and, on the other, having an impact that pushed the group towards adapting the new procedures.

### Being reasonable, but culturally sensitive

This sub-theme focuses on how the facilitator balanced being reasonable in relation to the implementation project, at the same time being as culturally sensitive to the group. In the interviews, the facilitator reflected on the different choices she had to make and, more specifically, her efforts to strike a balance between being a facilitator in an implementation process and being open to the concerns and emotions of a group of former colleagues. Even if research evidence indicated that the new procedures had advantages from the perspective of the patients and in terms of cost-effectiveness compared to the current procedures, each individual in the group tended to perceive the new procedures based on more personal perspectives. The facilitator felt a need to relate to these varying interpretations. One issue concerned the extent to which the new procedures would involve more work for the individual team member. This stage of implementing HBHC potentially included a shift in responsibility from the physician to the diabetes nurses. Among the physicians, this could be perceived as a means to relieve the pressure on them or as potentially losing control of the treatment, even though this would still be their medical responsibility. For the diabetes nurses, changes to procedures were likely to involve both more work and more responsibility, meaning that HBHC was met with some apprehension. One underlying concern that came up repeatedly in relation to the implementation process related to a chronic shortage of staff that had haunted the clinic for many years and was reputes to have had severe consequences on care and workplace morale.

As the implementation was performed at the facilitator’s previous place of work, she was aware of the different opinions in the group and of the existence of disagreement, hesitancy, as well as of consent and approval. The facilitator felt a strong need to take different opinions into consideration—whether expressed clearly or otherwise—and balance them against the implementation goals and the perspectives of children and families—actors who were not represented in the discussions. Paradoxically, as the facilitator’s familiarity with the culture and the people aided understanding when accommodating various interests, it also created tension and a sense of vulnerability in her role.

## Discussion

In this study we have shown that, to a large extent, the current literature on facilitation and change agency bypasses a number of critical elements related to the process of finding, taking and forming a role as facilitator. Recent reviews have indicated that successful facilitation or change agency is dependent on a wide variety of knowledge, skills and abilities, often presented in the form of lists of desirable qualities and conditions [9, 10, 18]. This study contributes significantly to these earlier findings by providing an in-depth example of a facilitation process described both from the perspective of the facilitator and as perceived by an accompanying research team conducting both interviews and observations of the implementation process and the facilitator’s actions.

The phenomenological hermeneutical method was chosen in order to elucidate the lived experience of being in a facilitator role. This method assumes that a phenomenon must be understood through original experiences of the world. Through, for example, narratives and reflection we are able to investigate and discover what is invariable in all the variations of a phenomenon, i.e. its essential meaning [29]. The method involves interpreting lived experience fixed in text. Interpretation takes place on the basis of preunderstanding and we cannot free ourselves from our preunderstanding; however, through critical reflection, we can revise, broaden and deepen our awareness. The individual researchers’ voices originated from different scientific perspectives, which helped the authors become aware of their preunderstanding, e.g. phenomena that were taken for granted. That one of the authors had multiple roles carried an obvious risk of bias and the result must be understood in relation to this circumstance. To strengthen the trustworthiness of the results, discussions with critical reflections on the different positions were carefully considered throughout the study. For the same reason, the first author with the facilitator role was excluded from part of the analysis.

During the analysis, four themes were identified, all of which involved balancing acts on different levels and in different ways. The theme that probably involved the most personally challenging aspects for the facilitator involved the balance between different roles, negotiating the role with the staff and coping with role-conflicts in this process. In this specific case, the facilitator depended on cooperation from the staff, met expectations from others and had to negotiate a number of different purposes and resources. The different roles included a background as a paediatric diabetes nurse (and a former colleague of some of the staff members), a key researcher and expert in the development and testing of the method, a part of the team that managed the implementation study and finally the role as facilitator to the staff involved in the implementation. The different roles bring with them different purposes, different resources and sources of power. Each of the roles also has its distinct ethos and pathos. The facilitator thus had to balance the researcher’s interest in evidence and scientific relevance with the realities of the clinical situation, with its various constraints in terms of time, resources and so on. Further complications were identified in the act of balancing the need to push ahead with the implementation project in the role of a key member of the research team against being more process oriented and consultative in a more facilitative role informed by models described in the literature [5, 31]. The different roles all involve many of the qualities described in the literature, but we show that it is not possible to add these desired qualities to each other in a linear fashion. Instead, they relate to specific roles that may very well be conflicting and actualize a need to strike a balance. While most studies of facilitation identify a facilitator’s qualities and skills in a decontextualized and atemporal perspective, this study provides insight into the impact of the facilitator’s previous relationship to the clinic and the different actors. This historical relationship cannot be reduced to a dichotomy of insider and outsider. Instead the facilitator is faced with the complex task of finding, taking and forming a role in a specific context as it has developed over time. For instance, the legitimacy, tacit knowledge and empathy that emerge from one’s professional experience as a nurse are powerful sources to draw from when finding authority in one’s role, yet may also provide experience, a pathos and an ethos that may be hard to reconcile with the priorities of the research project (i.e. pushing ahead with the implementation) or being more process oriented and, in that case, even challenging assumptions held by the staff.

We claim that these balancing acts, in which the facilitator struggles to identify what Reed and Bazalgette [15] describe as a “regulating principle inside oneself which enables one, as a person, to manage one’s behaviour in relation to what needs to be done to further the purpose of the system within which the role is to be taken” (p. 4), are a key component in finding, taking and shaping an efficient role as facilitator. Furthermore, an important finding in this study is that issues that come close to one’s professional and personal identity are involved in a profound manner and that facilitators run the risk of underestimating—or even avoiding—the need to make expectations more explicit and to negotiate their role. This is probably especially important when establishing collaboration with key insiders (clinical champions and formal and informal leaders). As Krantz and Maltz [16] suggest, these negotiations probably have to involve both formal and more informal role expectations and how the role is given and taken. They argue that an individual’s contribution and effectiveness in an organization can be understood only as a function of how well the individual and the organization negotiate the boundary between the role as given—which constitutes the organization’s expectations—and the role as taken—how the role is taken on held internally. This negotiation or alignment is further complicated by the task and the sentient systems operating within the organization. In order for the facilitator to contribute in an effective way and act with authority in the role, it thus seems that awareness of the different roles—and what they bring with them—and early and possibly iterative opportunities for reflection and negotiation of expectations can be helpful.

We also showed that striking a balance can have consequences that are sometimes surprising and frustrating. This is demonstrated in the theme concerning the facilitator’s sense of having all the necessary qualifications and still experiencing of inadequacy. An implication of this finding is that emotions and affects are important indicators that, if used as feedback, can be a powerful tool that helps to manage the facilitation process and shape the facilitator role.

A more instrumental aspect of the balancing the plethora of possible roles is the implications this has on the more specific styles of leadership or facilitation adopted by the facilitator. As we showed in the theme concerning a controlling versus more permissive style of leadership, there is a need to identify a proper balance between a style that involves setting the agenda and moving towards specific goals, versus a more participatory style of leadership. The theme related to leadership styles forms part of a wider discussion on the balance between managing the implementation process in a specific direction, versus providing opportunities for reflection, learning and the questioning of assumptions that may lie in the way of new ways of working. The facilitator has to strike a balance between following a plan or a recipe versus creating a space that allows for double loop learning and critical reflection. The limitations of programmatic, planned or linear modes of organizational change have also been a theme in recent studies of change processes, in which reflection, questioning of assumptions are prominent elements [11, 12]. Finally, on what could possibly be described as a more fundamental level, we identified a theme concerning balancing different values—being reasonable, but culturally sensitive. Balancing values involves the facilitator in issues concerning not only empathy for those involved in the implementation process, but also the ethics of research and of care in a more profound sense.

This study adds to the literature as we show that it is not sufficient to add up the desirable qualities of a facilitator in a linear and cumulative way. Instead, knowledge, skills and abilities within distinct roles in a specific context in which the roles may be in conflict and cause frustration are experienced as a balancing act. The main implication is that the experience of being a facilitator can be more fully described by applying the concept of role, thus allowing reflection and analysis to take place in a more holistic way, which in turn provides a better foundation from which to negotiate the facilitator role.

## Abbreviations

HBHC: Hospital-based Home Care
MRC: Medical Research Council
